# Acute Physical, Mental and Sequential Combined Fatigue Protocols Differentially Affect Basketball Shooting and Psychomotor Vigilance: A Randomized Crossover Study

**DOI:** 10.3390/life16101620

**Published:** 2026-09-26

**Authors:** Burak Karaca, Mustafa Özdal, Fatma Beyza Bilgiç, Ahmet Koyunlu, Murat Bilgiç, Ali Yıldırım

**Affiliations:** 1 Department of Physical Education and Sports, Graduate School of Health Sciences, Gaziantep University, 27350 Gaziantep, Türkiye; 2 Department of Coaching Education, Faculty of Sports Science, Gaziantep University, 27350 Gaziantep, Türkiye; 3 Faculty of Sport Sciences, Physical Education and Sport, Batman University, 72000 Batman, Türkiye; fatmabeyza.bilgic@batman.edu.tr (F.B.B.); murat.bilgic@batman.edu.tr (M.B.); 4 Department of Coaching Education, Faculty of Sport Sciences, Kahramanmaraş Sütçü İmam University, 46100 Kahramanmaraş, Türkiye; koyunluahmet@gmail.com; 5 School of Physical Education and Sport, Kilis 7 Aralık University, 79000 Kilis, Türkiye; ali.yildirim@kilis.edu.tr

**Keywords:** basketball, mental fatigue, physical fatigue, combined fatigue protocol, shooting accuracy, psychomotor vigilance, multiple-object tracking, crossover study

## Abstract

Basketball shooting and attention may respond differently to cognitive and physical demands. We compared isolated and sequential combined fatigue protocols within the same players. Twelve male players completed a randomized four-period crossover: documentary control, 30 min AX Continuous Performance Task (AX-CPT), 90 s maximal burpees, and AX-CPT followed by burpees. Performance models accounted for participant, period, sequence and applicable baselines. False discovery rate correction used three outcome domains. Combined reduced dynamic shooting accuracy, relative to control (odds ratio 0.53, 95% confidence interval (CI) 0.41–0.67; Holm *p* < 0.001) and mental (0.66, 0.52–0.85; Holm *p* = 0.004). Compared with control, combined increased brief psychomotor vigilance test reaction time by 23.69 ms (95% CI 7.10–40.27; Holm *p* = 0.027) and reduced application-reported accuracy by 12.39 percentage points (95% CI 6.22–18.57; Holm *p* < 0.001). Mental-versus-control vigilance contrasts were also significant. Free-throw and vigilance variability findings were model-sensitive. Tracking, physiological and neuromuscular outcomes, and factorial interactions, were non-significant after correction. The sequential AX-CPT-then-burpee protocol was associated with lower dynamic shooting accuracy. Mental and combined protocols were associated with poorer end-of-session vigilance.

## 1. Introduction

Basketball requires repeated high-intensity movement alongside sustained attention, rapid decisions and accurate technical execution [1,2]. Shooting depends on coordinated force transfer, release mechanics and visual information [3,4]. Understanding how mental and physical fatigue protocols influence shooting is therefore relevant to performance assessment, particularly when cognitive and physical demands occur in close succession.

Mental fatigue commonly describes tiredness and reduced energy associated with sustained cognitive demand [5,6]. Physical fatigue is considered here in the context of muscular exercise, encompassing reported tiredness and possible decrements in physical performance [7]. In this study, the mental condition involved the cognitively demanding AX Continuous Performance Task (AX-CPT), the physical condition involved burpee exercise, and the combined condition involved AX-CPT followed by burpees. These labels describe the predominant demands and sequence of the experimental tasks, not distinct fatigue entities or isolated physiological mechanisms. Subjective fatigue responses and measured performance are evaluated separately.

Basketball studies have reported altered shooting and attention after physical exercise or demanding cognitive activity, although findings vary with the protocol and player characteristics [5,7,8,9,10,11,12,13,14,15]. Within-sample comparisons of cognitive, physical and combined tasks have already been conducted [16]. Mortimer and colleagues also used a sequential combined protocol comprising a 15 min time-load dual-back task followed by 90 s burpees to examine throwing and endurance performance [17]. Tasks with different cognitive and physical demands may elicit different subjective fatigue responses and influence subsequent performance. Comparing their separate and sequential administration, therefore, addresses how responses vary across task protocols. The Settings, Protocol establishments, Confounders, Individuals, Framework and Yield (SPeCIFY) guidelines describe cognitive and physical tasks according to their predominant demands, and emphasize reporting task properties and combining subjective and behavioural assessment when characterizing mental fatigue [18].

A specific question in basketball is whether prolonged cognitive demand followed by brief, intense exercise alters shooting relative to either protocol alone. Because recovery after intense exercise is time-dependent [19], the order and duration of sequential tasks are relevant to interpreting their performance effects.

This study compared dynamic shooting and free-throw performance following cognitive, physical, and sequential combined-task protocols intended to elicit fatigue, with documentary viewing as the control condition, in the same basketball players. Subjective fatigue ratings and cognitive, cardiovascular, and handgrip outcomes were assessed to characterize the responses associated with each protocol. We hypothesized that shooting performance would be poorest after the combined protocol and that psychomotor vigilance would be lower after the mental and combined protocols than after control. A factorial analysis examined the mental-task-by-physical-task interaction. Subjective mental and physical fatigue ratings characterized responses before the assigned protocol, immediately afterward and at the end of the experimental session.

## 2. Materials and Methods

### 2.1. Study Design

A controlled, randomized, four-condition, four-period, within-participant crossover design was used. Each participant completed control, mental, physical and sequential combined fatigue protocols. Reporting was guided by Consolidated Standards of Reporting Trials (CONSORT) 2025 and the extension for randomized crossover trials [20,21], with task and statistical reporting informed by SPeCIFY and Checklist for Statistical Assessment of Medical Papers (CHAMP) [18,22]. Experimental sessions took place on the participating clubs’ indoor basketball courts in İzmir, Türkiye, between June and August 2026. Each player completed four experimental sessions over approximately three weeks, with at least 48 h between visits.

### 2.2. Participants

**Figure 1:**
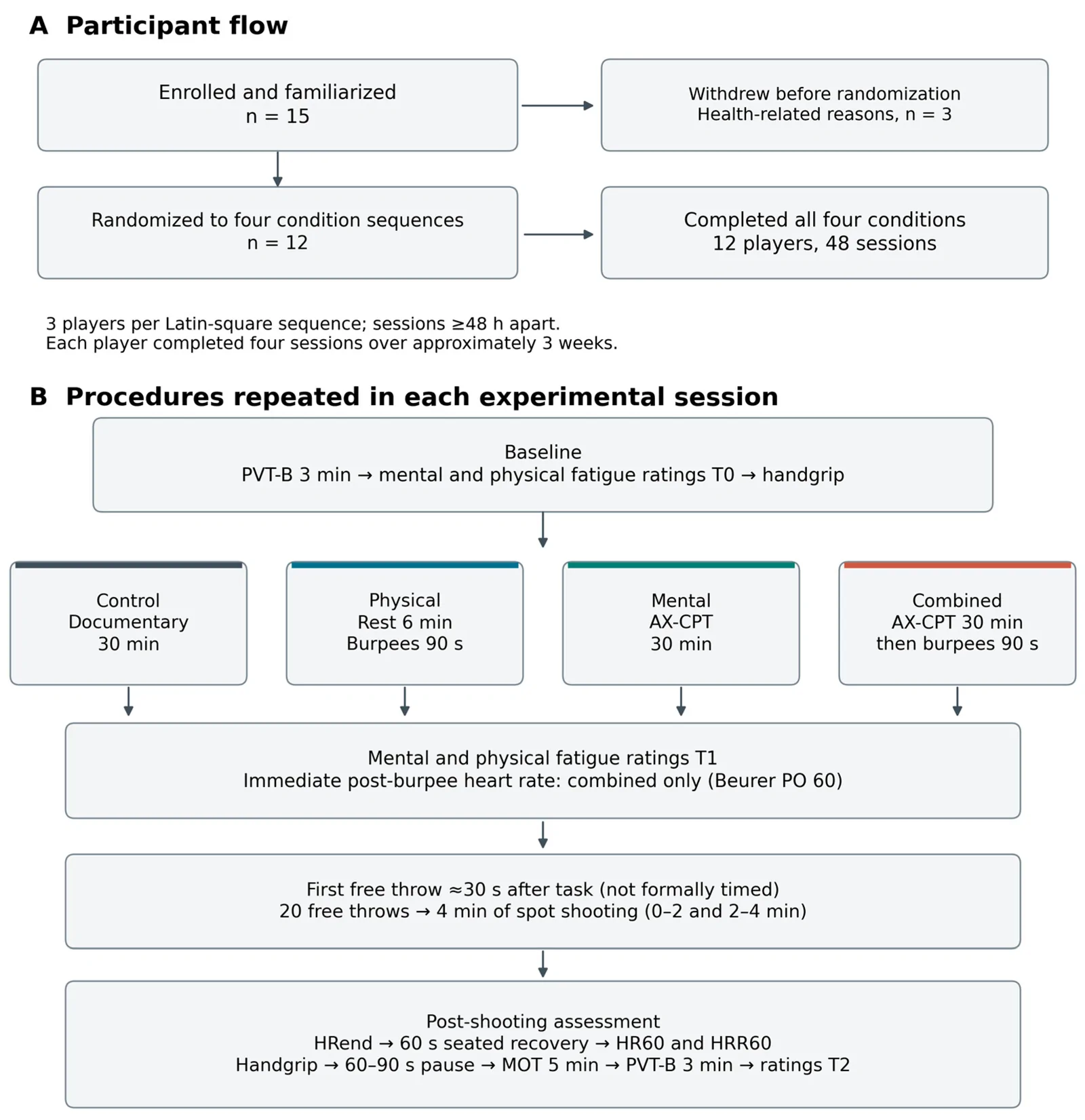
Participant flow and experimental session procedures. (**A**) shows enrolment, allocation and completion; (**B**) shows the task conditions and assessment sequence. Arrows indicate procedural order. AX Continuous Performance Task (AX-CPT); brief psychomotor vigilance test (PVT-B); multiple-object tracking (MOT); heart rate immediately after shooting (HRend); heart rate after 60 s of seated recovery (HR60); heart-rate recovery over 60 s (HRR60). Assessment times: before the assigned protocol (T0); immediately after the protocol (T1); after the assessment battery (T2).

Fifteen male basketball players were enrolled and attended familiarization. Three withdrew for health-related reasons after familiarization and before randomization; twelve were randomized and completed all four conditions (Figure 1). Players were recruited from three clubs competing in the Turkish Men’s Regional Basketball League. Competition level, basketball experience and training context characterized the sample [23]. Age and anthropometric characteristics are summarized in Table 1. All players had at least eight years of basketball experience and no health condition preventing completion of the procedures. Coaches reported summer training on at least five days per week and characterized typical session intensity as a rating of perceived exertion (RPE) of approximately 5–6. This information describes the team training context. Participants received information about the procedures, risks, benefits, confidentiality and right to withdraw, and provided written informed consent.

Sample size was determined a priori in G*Power 3.1 (Heinrich Heine University, Düsseldorf, Germany) [24,25] for four-minute made shots (MAKE_4_MIN). Planning was informed by Daub et al.’s shooting study [15], using f = 0.40 as a large-effect assumption. The F-test option “Analysis of variance (ANOVA): Repeated measures, within factors” used α = 0.05, power = 0.80, one group, four measurements, an assumed repeated-measures correlation of 0.50 and nonsphericity correction ε = 1.00, yielding a minimum of 10 participants. Fifteen players were enrolled to allow for losses. The calculation targeted the omnibus condition comparison for MAKE_4_MIN.

### 2.3. Randomization and Standardization

The lead researcher generated four cyclic Latin-square condition sequences. An independent researcher prepared 12 opaque sealed envelopes, with three envelopes containing each sequence. The envelopes were shuffled and randomly distributed to participants; each participant’s envelope was opened before his first experimental session. Three players were allocated to each sequence: control–physical–mental–combined; physical–mental–combined–control; mental–combined–control–physical; and combined–control–physical–mental. All 48 sessions followed the assigned order, with each condition occurring three times in each period. A period denotes the participant’s first-through-fourth experimental visit, excluding familiarization. Participants and administrators were aware of the tasks. Shooting and device-derived data were coded, and condition labels were masked during data cleaning and the initial analysis.

Participants attended one familiarization session covering the subjective rating scales, brief psychomotor vigilance test (PVT-B), multiple-object tracking (MOT), handgrip assessment, AX-CPT and shooting protocol. They were asked to avoid caffeine, nicotine, alcohol and strenuous exercise for 24 h before each session; compliance was confirmed verbally on arrival. They were also instructed to maintain their usual sleep and dietary routines. The same ball type, court layout, rebounder/passer, devices, instructions and outcome order were used across conditions. Sessions were conducted in an approximately 09:00–12:00 morning window, with repeated visits scheduled at approximately the same time of day for each participant.

### 2.4. Experimental Session and Fatigue Protocols

Each session began with a 3 min PVT-B, baseline mental and physical fatigue ratings (T0), and bilateral isometric handgrip assessment. The assigned protocol was then administered, immediately followed by post-task subjective ratings (T1). Participants next completed the standardized shooting test (SST), after which heart rate was recorded at the end of the test and again after 60 s of seated recovery. Post-session handgrip testing, a 60–90 s pause, a 5 min MOT task, and a second 3 min PVT-B followed. Final mental and physical fatigue ratings were recorded at the end of the session (T2).

In the control condition, all participants watched the same 30 min Turkish-dubbed excerpt from Our Great National Parks (2022). This fixed documentary standardized the audiovisual content across participants.

The mental protocol comprised a 30 min AX-CPT on SOMA NPT (version 15.0; sswitch Neuro Performance Solutions Limited, Luzern, Switzerland) using an 11th-generation iPad (Apple Inc., Cupertino, CA, USA). AX-CPT has been used as a demanding cognitive task in mental-fatigue research [6]. Task duration was the only user-selectable setting, and was set to 30 min. The task contained AX, AY, BX and BY cue–probe sequences. Participants selected the left response only when a red A was followed by a red X, selected the right response for red AY, BX and BY sequences, and ignored all white letters. The task involved context maintenance, response selection and performance monitoring [26,27]. The same device and task configuration were used across sessions. Whole-task reaction time, accuracy, lapse count and coefficient of variation (CV) were recorded directly from the application.

The physical protocol comprised 90 s of maximal burpees with standardized verbal encouragement, preceded by a 6 min passive interval after baseline handgrip assessment. The combined protocol comprised 30 min of AX-CPT followed immediately by 90 s of maximal burpees. The AX-CPT preceded the burpee task to avoid placing the 30 min cognitive task between physical exercise and shooting assessment. Task-phase durations were approximately 30 min for control and mental, 7.5 min for physical including pre-exercise rest, and 31.5 min for combined.

Participants proceeded directly from the post-task assessment to shooting without a planned recovery period. The first free throw generally began approximately 30 s after task completion; this interval was not formally timed. All 20 free throws preceded the four-minute dynamic shooting section. In the combined condition, heart rate was measured with the Beurer PO 60 pulse oximeter (Beurer GmbH, Ulm, Germany) immediately after the 90 s burpee task.

### 2.5. Outcome Measures

#### 2.5.1. Subjective Mental and Physical Fatigue

Participants rated mental and physical fatigue separately at T0, T1 and T2, using study-specific 1–10 numerical scales informed by Borg’s scaling approach [28]. The questions were “How physically fatigued do you feel right now?” and “How mentally fatigued do you feel right now?”, each rated from 1 to 10. The endpoints were 1, the lowest fatigue level, and 10, the highest fatigue level. T1 captures the immediate subjective response to the assigned task, whereas T2 reflects the cumulative session, including shooting and subsequent assessments. The two items describe reported sensations, and do not establish distinct fatigue mechanisms.

#### 2.5.2. Adapted Standardized Shooting Test

The shooting protocol was adapted from the standardized shooting task developed by Daub and colleagues [29]. Participants first attempted 20 consecutive free throws, each within 5 s of receiving the pass. They then completed 4 min of spot-to-spot shooting from seven marked positions, all set 4.57 m from the basket. Starting at the left baseline, the player advanced after making two shots at the current position; the two makes did not need to be consecutive. The sequence was 1–2–3–4–5–6–7–7–6–5–4–3–2–1 and continued for the full 4 min. Two balls were used, dribbling was not permitted, and attempts taken outside the marked position or while touching the line were recorded as misses.

Dynamic shooting accuracy (100 × made shots/total attempts) and free-throw accuracy (100 × makes/20) represented shooting success. MAKE_4_MIN and total attempts characterized shooting output; misses and completed spots were descriptive because they are mathematically or procedurally dependent on makes and attempts. Session-level counts were recorded for the first and last 10 free throws and for the 0–2 and 2–4 min dynamic-shooting intervals. Completed spots were calculated as the integer part of total makes divided by two, with progression continuing across the 2 min boundary. Compared with the original protocol, the adaptation reduced free throws from 60 to 20, set all positions at 4.57 m, and required two total, rather than two consecutive, makes before advancing [29].

#### 2.5.3. Cognitive Performance

The 3 min PVT-B was administered before the task and at the end of the session. Mean reaction time and application-reported accuracy were the principal vigilance measures; lapse count and CV provided additional information about the response pattern. SOMA classifies responses ≥355 ms as lapses, and excludes those latencies from its reported mean reaction time [30]. The lapse threshold originates from PVT-B validation work [31]. Lapse count therefore captures delayed responses separately from the reported mean latency.

Accuracy denotes the percentage of responses classified as correct by SOMA NPT, and CV denotes the application-reported coefficient of variation in response time [32]. Reaction time, accuracy, lapses and CV were recorded directly from SOMA NPT. Each application output was analysed separately.

The SOMA NPT task labelled MOT was administered for 5 min near the end of each session on the same iPad. A designated target was tracked among several moving objects and selected, after which another target was presented. Targets were followed successively during the task. Application-reported mean reaction time, accuracy and CV were recorded directly, and analysed as exploratory cognitive outcomes.

#### 2.5.4. Cardiovascular Outcomes

A Beurer PO 60 pulse oximeter was applied immediately after the SST while the participant assumed a standardized seated position. HRend was the first stable heart-rate value displayed for approximately 3 s, and HR60 was recorded at exactly 60 s. Post-shooting heart-rate recovery was calculated as HRR60 = HRend − HR60 (beats·min^−1^). Peripheral oxygen saturation (SpO_2_) was recorded once per session after the SST, for safety assessment and exploratory analysis. An SpO_2_ value below 92% was the criterion for terminating the session.

Immediate post-burpee heart rate in the combined condition was summarized descriptively, as an indicator of cardiovascular load.

#### 2.5.5. Isometric Handgrip Control

Handgrip performance was assessed with a Camry EH101 digital dynamometer (Zhongshan Camry Electronic Co., Ltd., Zhongshan, China) using a standardized seated position: the shoulder near the trunk, elbow at approximately 90°, and forearm and wrist neutral. Standardized handgrip positioning and the reliability of handgrip measurement in basketball players have been described previously [33,34,35]. At the beginning of each session, participants completed two 3 s maximal voluntary contractions per hand, separated by 60–90 s; the higher value defined that hand’s session-specific maximal voluntary contraction (MVC). Participants then attempted to maintain a recorded target of approximately 50% MVC for 30 s, with visual feedback. The same absolute target was used for the pre- and post-session tests, and MVC was re-established at the next experimental visit. The dominant hand was tested first, followed by at least 90 s of rest and the non-dominant hand.

The dynamometer display was recorded with an iPhone 15 Pro (Apple Inc., Cupertino, CA, USA) at 4K resolution and 60 frames·s^−1^. Videos were coded to mask condition and pre/post measurement status. Two assessors independently extracted one force value per second and then compared their readings. Every extracted value agreed, and one final force series was retained. The endurance ratio was the mean force during the final 10 s divided by the mean during the first 10 s. Stability error was 100 × Σ|force − target force|/(target force × 30). These indices characterize maintenance of force and deviation from the target during the sustained contraction [36,37].

### 2.6. Statistical Analysis

Statistical analyses were performed using Python 3.12.14, NumPy 2.3.5, pandas 2.2.3 and SciPy 1.17.0. The participant was the independent experimental unit. Descriptive values are participant-level means and standard deviations (SDs), with t-based 95% confidence intervals (CIs), where indicated. All 12 players had complete observations for each applicable outcome. Inference was exploratory and organized by outcome domain.

Dynamic shooting and free throws were modelled as aggregated successes and failures using a binomial distribution with a logit link, fixed effects for condition, categorical period (1–4) and assigned sequence, and a normally distributed participant random intercept. The denominator was the observed number of dynamic attempts or 20 free throws. Overall condition effects were assessed by likelihood-ratio tests against models retaining period, sequence and the random intercept. Period and sequence remained in the models throughout, and were assessed as design diagnostics. Conditional odds ratios (ORs) and approximate 95% Wald CIs were estimated for condition contrasts. Beta-binomial mixed models assessed extra-binomial variation; linear models of participant-session percentages provided percentage-point estimates. Participant fixed-effects models with period provided F tests and t-based contrast intervals, absorbing sequence into participant effects. Dynamic-shooting paired contrasts also used exact sign-flip tests over 4096 sign combinations, assuming symmetric paired differences.

PVT-B post-session outcomes used linear mixed models with condition, period, sequence, the corresponding session-specific baseline standardized to a z score, and a participant random intercept. Handgrip outcomes used the same baseline-adjusted structure. Other outcomes in these three domains used the crossover model without a baseline term. Maximum likelihood was used for overall likelihood-ratio tests; restricted maximum likelihood provided linear-model estimates and approximate Wald CIs. Participant fixed-effects models with period and the applicable baseline provided finite-sample sensitivity tests and t-based intervals. A fractional-logit sensitivity model for PVT-B accuracy included condition, period, sequence and baseline, accommodating boundary values [38]. Participant-cluster delete-one jackknife standard errors and t-based intervals with 11 degrees of freedom accounted for repeated visits; contrasts were tested on the logit scale. Gaussian models of count and bounded summaries were treated as approximations, and interpreted alongside the sensitivity results.

The four conditions were also parameterized as mental-task absent/present × physical-task absent/present for dynamic shooting accuracy, PVT-B reaction time, PVT-B accuracy and MOT reaction time. Models retained period, sequence and the applicable PVT-B baseline. The shooting interaction was tested by comparing binomial models with and without the interaction; its ratio of odds ratios was exp(βCombined − βMental − βPhysical), with control as reference. An analogous contrast described interaction on the percentage-point scale. Linear contrasts tested the cognitive interactions. These analyses assessed departures from additivity on their respective fitted scales.

Benjamini–Hochberg false discovery rate (FDR) control at 0.05 was applied separately to three families of overall condition tests: basketball performance (dynamic accuracy, free-throw accuracy, MAKE_4_MIN and attempts; four tests); cognitive performance (PVT-B reaction time, accuracy, lapses and CV, plus MOT reaction time, accuracy and CV; seven tests); and physiological/neuromuscular outcomes (HRend, HR60, HRR60, SpO_2_, and dominant/non-dominant handgrip endurance ratio and stability error; eight tests). The four factorial interaction tests formed a separate FDR family. A pooled correction across all 19 condition tests and the same domain corrections applied to finite-sample sensitivity tests assessed dependence on the inferential framework. The six pairwise condition contrasts within each outcome/model in these domains were Holm-adjusted. CIs were pointwise-, and not multiplicity-, adjusted. Primary model results and sensitivity results are reported separately.

Mental and physical fatigue ratings were analysed separately using 4-condition × 3-time repeated-measures ANOVAs, with T0, T1 and T2 treated as repeated observations. Greenhouse–Geisser corrections were applied to the degrees of freedom of each within-subject effect; effect sizes were expressed as partial eta squared (η_p_^2^). The two condition-by-time interactions were the principal subjective-response tests and were Holm-adjusted across rating domains. Condition and time main effects were supporting tests. Follow-up paired t tests compared conditions at each time (18 comparisons per rating domain) and times within each condition (12 comparisons per domain), with Holm adjustment separately across each of these sets. Sensitivity analyses evaluated T1 − T0 and T2 − T0 changes using participant fixed effects, categorical period and condition; sequence was absorbed by participant effects. Their four overall condition tests were Holm-adjusted together, and six pairwise contrasts were Holm-adjusted within each model. Paired change-score contrasts also used Holm adjustment across six comparisons per domain and change interval. Contrast CIs were pointwise t-based 95% intervals. Whole-task AX-CPT summaries were descriptive. Binomial integration used 81-point Gauss–Hermite quadrature, checked against 121 points. All contrast tests were two-sided.

## 3. Results

### 3.1. Shooting Performance

**Figure 2:**
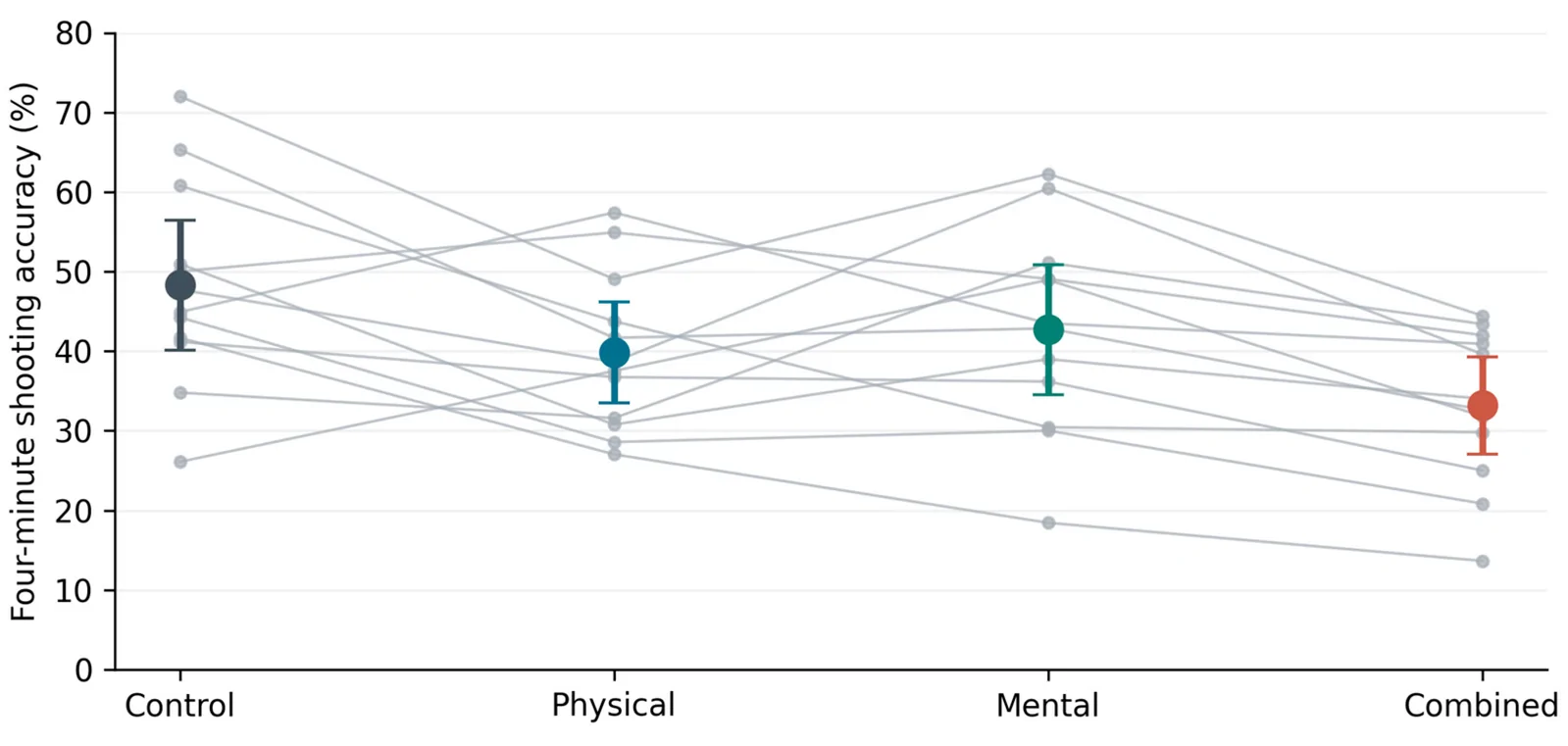
Individual four-minute shooting percentages. Thin lines connect repeated observations from the same player; the displayed condition order is not chronological session order. Large markers show means and t-based 95% confidence intervals across 12 players. Percentages were calculated for each player before averaging.

All 12 randomized players completed the four conditions, yielding 48 sessions. Mean four-minute shooting accuracy was 48.30% in control, 39.80% after the physical protocol, 42.68% after the mental protocol and 33.17% after the combined protocol (Table 2; Figure 2). The period- and sequence-adjusted binomial model detected an overall condition difference (likelihood-ratio χ^2^(3) = 29.09, *p* < 0.001; FDR q < 0.001). Accuracy was lower after combined than control (OR 0.53, 95% CI 0.41–0.67; Holm *p* < 0.001) and mental (OR 0.66, 95% CI 0.52–0.85; Holm *p* = 0.004; Table 3).

Adjusted percentage-point differences for combined versus control and combined versus mental were −15.13 (95% CI −21.84 to −8.42; Holm *p* < 0.001) and −9.51 (95% CI −16.22 to −2.80; Holm *p* = 0.027), respectively. Both contrasts remained significant in the beta-binomial model (Holm *p* < 0.001 and *p* = 0.012) and the participant fixed-effects percentage model with period (Holm *p* = 0.001 and *p* = 0.047). Paired exact sign-flip tests also supported these comparisons (Holm *p* = 0.032 and *p* = 0.003). Physical versus control was significant in the binomial and beta-binomial models, but not in adjusted percentage or paired analyses. Combined versus physical was not significant after Holm correction in these adjusted models.

MAKE_4_MIN differed across conditions (χ^2^(3) = 15.13, *p* = 0.002; basketball-family q = 0.003). The adjusted combined-versus-control difference was −7.25 made shots (95% CI −10.89 to −3.61; Holm *p* < 0.001). The remaining made-shot contrasts did not reach significance after Holm correction.

Free-throw accuracy showed an overall condition effect in the binomial model (χ^2^(3) = 9.70, *p* = 0.021, q = 0.028). Combined was lower than control (OR 0.59, 95% CI 0.41–0.85; Holm *p* = 0.026). This contrast did not reach significance in the beta-binomial model (Holm *p* = 0.081) or the participant fixed-effects percentage model (difference −12.92 points, 95% CI −24.53 to −1.31; Holm *p* = 0.182). The finite-sample overall percentage test gave F(3,30) = 2.03, *p* = 0.131.

The mental-task-by-physical-task interaction was not significant on the log-odds scale (ratio of odds ratios 0.95, 95% CI 0.68–1.33; *p* = 0.766; interaction-family q = 0.895). The adjusted percentage-scale interaction was −1.01 points (95% CI −10.50 to 8.48; *p* = 0.835). Period and sequence diagnostic tests gave *p* = 0.111 and 0.089, respectively. Mean total attempts ranged from 48.00 to 48.42.

### 3.2. Psychomotor Vigilance

Baseline-, period- and sequence-adjusted condition tests were significant for PVT-B reaction time (χ^2^(3) = 12.29, *p* = 0.006, cognitive-family q = 0.020) and application-reported accuracy (χ^2^(3) = 18.50, *p* < 0.001, q = 0.002; Table 4). Mental and combined protocols were associated with longer reaction times and lower accuracy than control (Table 5). Participant fixed-effects analysis of covariance (ANCOVA) supported both control comparisons for each outcome. Fractional-logit sensitivity estimates for accuracy were −1.12 (95% CI −1.67 to −0.58; Holm *p* = 0.005) for mental versus control and −1.54 (95% CI −2.49 to −0.58; Holm *p* = 0.023) for combined versus control on the logit scale. Period and sequence diagnostic *p* values were 0.874 and 0.331 for reaction time, and 0.849 and 0.904 for accuracy.

**Figure 3:**
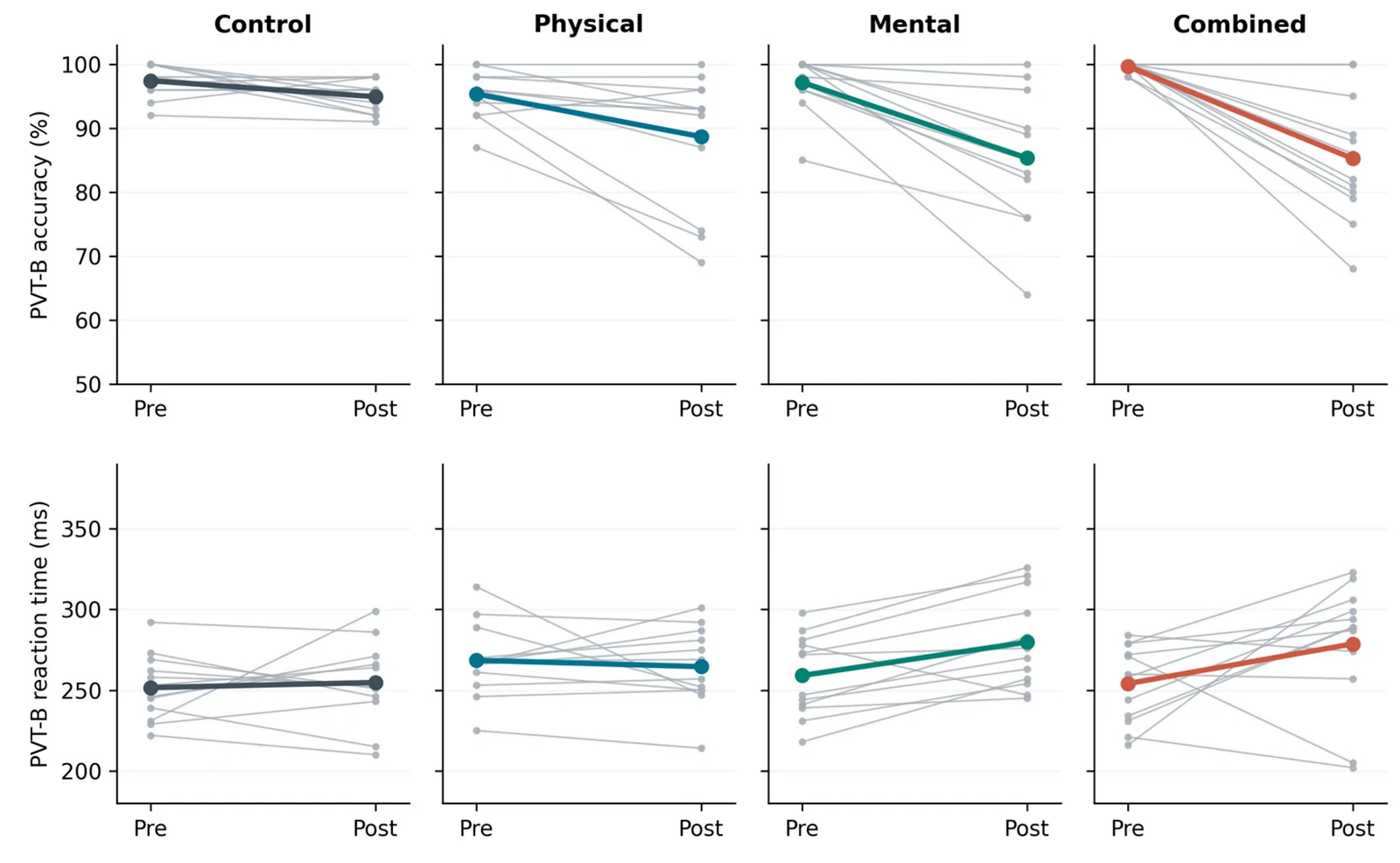
Individual PVT-B pre-to-post trajectories by condition. Thin lines represent players and thick coloured lines represent condition means. Post-test values were obtained at the end of the assessment battery. The figure shows observed values; baseline-adjusted contrasts are reported in Table 5.

Combined-versus-control differences were 23.69 ms (95% CI 7.10–40.27; Holm *p* = 0.027) for reaction time and −12.39 percentage points (95% CI −18.57 to −6.22; Holm *p* < 0.001) for accuracy. Mental-versus-control differences were 24.32 ms (95% CI 7.57–41.07; Holm *p* = 0.027) and −9.28 percentage points (95% CI −15.17 to −3.40; Holm *p* = 0.010), respectively. Interactions were not significant for reaction time (−8.93 ms, 95% CI −33.46 to 15.60; *p* = 0.476) or accuracy (0.62 percentage points, 95% CI −8.54 to 9.77; *p* = 0.895; both q = 0.895). Figure 3 shows individual trajectories.

PVT-B lapses and CV showed overall condition effects in the primary models (*p* = 0.011 and *p* = 0.012, respectively; both cognitive-family q = 0.020; Table 4). Compared with control, the combined protocol was associated with more lapses (difference 5.64, 95% CI 1.99–9.28; Holm *p* = 0.015) and greater reaction-time variability (CV difference 26.42 percentage points, 95% CI 9.29–43.55; Holm *p* = 0.015). The participant fixed-effects contrasts gave Holm *p* = 0.018 and *p* = 0.035, respectively. Under domain-wise FDR correction of the finite-sample overall tests, lapses remained significant (q = 0.041), whereas CV did not (q = 0.064).

### 3.3. Multiple-Object Tracking

**Figure 4:**
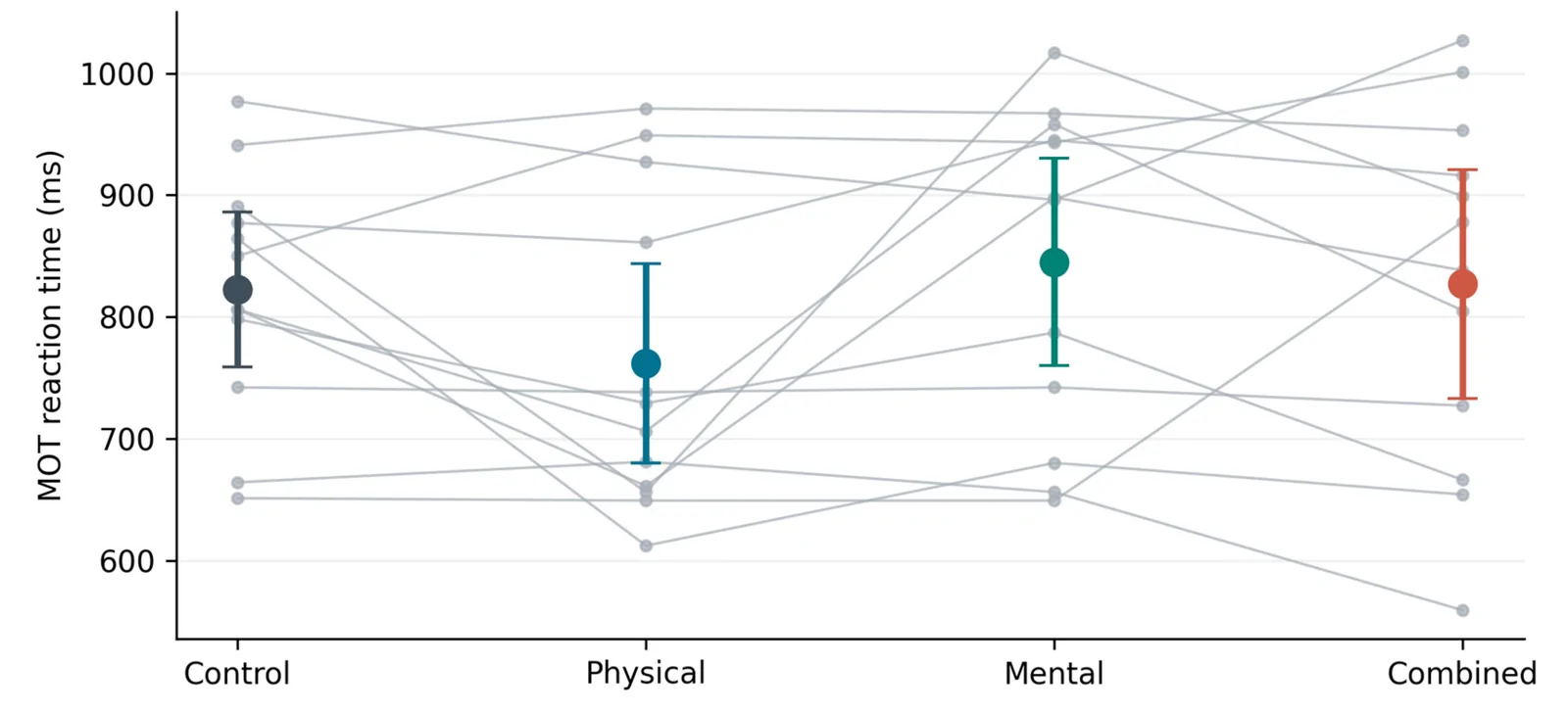
Individual post-session MOT reaction times. Thin lines connect observations from the same player in display order, not chronological visit order. Large markers show condition means with pointwise t-based 95% confidence intervals. The plot displays observed session summaries; adjusted contrasts are reported in Table 6.

MOT reaction time, accuracy and CV did not reach significance after cognitive-family FDR correction (all q = 0.060; Table 4). For reaction time, the unadjusted overall test was χ^2^(3) = 7.89, *p* = 0.048, and none of the six Holm-adjusted pairwise contrasts was significant (Table 6; Figure 4). The largest contrast was mental versus physical: 83.17 ms (95% CI 19.10–147.23; Holm *p* = 0.066). The participant fixed-effects sensitivity test gave F(3,30) = 2.45, *p* = 0.083, and the corresponding mental-versus-physical contrast gave Holm *p* = 0.098. The pooled 19-test q value for reaction time was 0.114. Its mental-task-by-physical-task interaction was 42.67 ms (95% CI −47.93 to 133.27; *p* = 0.356; interaction-family q = 0.895). Period and sequence diagnostic *p* values were 0.432 and 0.242.

### 3.4. Subjective Fatigue and Task Responses

Condition-by-time interactions were significant for mental ratings (F(3.39, 37.31) = 32.97, ε = 0.565, partial η_p_^2^ = 0.750) and physical ratings (F(3.95, 43.41) = 11.36, ε = 0.658, partial η_p_^2^ = 0.508; both Holm *p* < 0.001). At T1, mental ratings were higher than control after the mental protocol by 4.67 points (95% CI 3.28–6.06) and after combined by 5.17 points (4.20–6.14). Physical ratings were higher than control after the physical protocol by 3.17 points (2.27–4.06) and after combined by 4.25 points (2.95–5.55; all four Holm *p* < 0.001). Physical ratings did not differ significantly between combined and physical at T1 or T2 (Holm *p* = 0.753 and 0.311, respectively).

**Figure 5:**
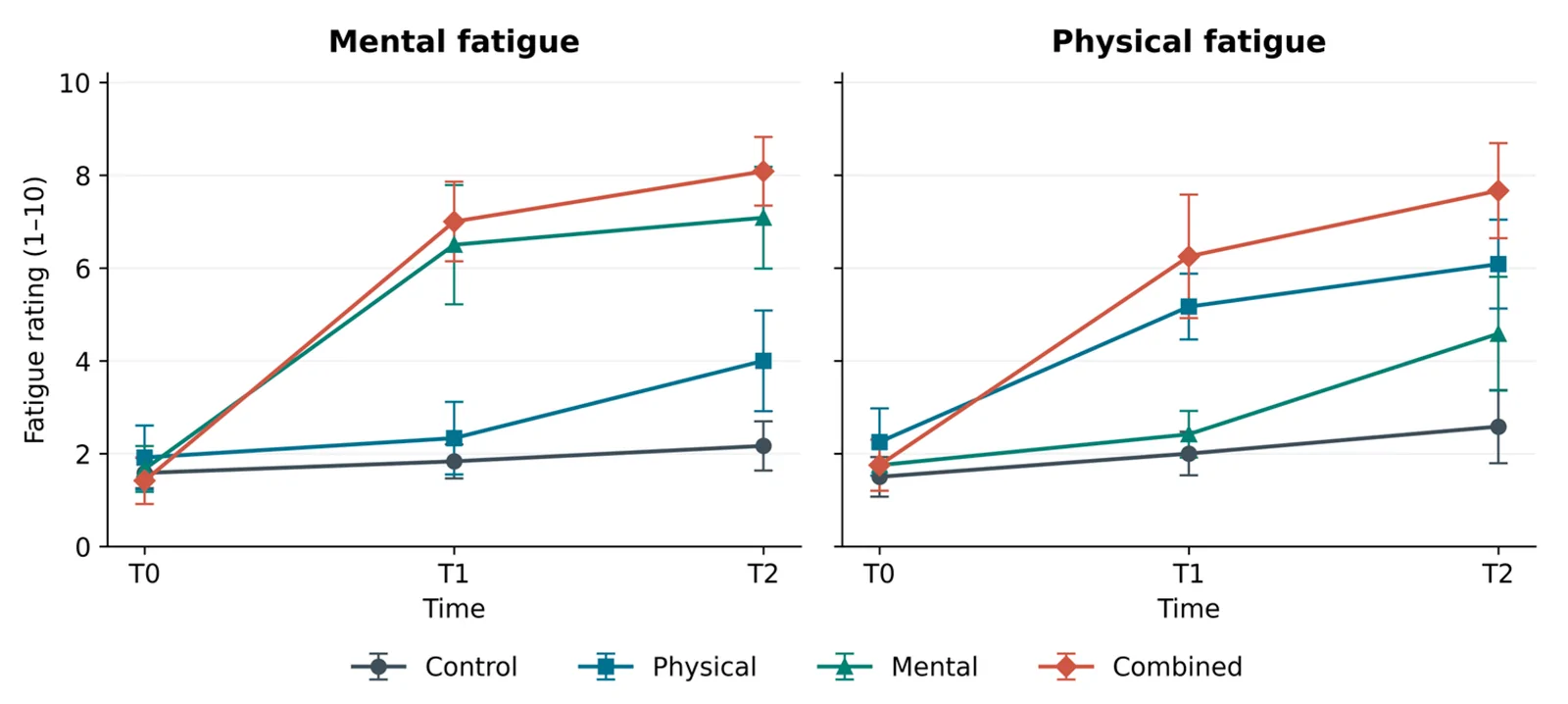
Mental and physical fatigue ratings across the experimental session. Points and vertical bars show means and pointwise 95% t-based confidence intervals for 12 players. T0, before the assigned protocol; T1, immediately after the protocol; T2, after the full assessment battery. Ratings used study-specific 1–10 scales. Lines connect condition means across the three assessment times; condition definitions are given in Table 2.

From T1 to T2, mental ratings increased after the physical protocol by 1.67 points (95% CI 0.64–2.69; Holm *p* = 0.030), and physical ratings increased after the mental protocol by 2.17 points (1.06–3.28; Holm *p* = 0.009). At T2, mental ratings were higher after combined than mental by 1.00 point (0.39–1.61; Holm *p* = 0.035). Period-adjusted change analyses supported overall condition differences in both rating domains at both post-task times (all Holm *p* < 0.001). The combined-versus-physical contrast in physical change scores was model-sensitive. T2 followed the complete assessment battery. Table 7 gives descriptive values and indicates significant condition and time comparisons; Figure 5 shows observed trajectories.

For mental and combined conditions, respectively, whole-task AX-CPT reaction time was 538.33 ± 102.20 and 628.25 ± 160.23 ms, accuracy 82.42 ± 9.89% and 80.50 ± 8.17%, lapse count 4.92 ± 2.54 and 7.25 ± 4.56, and CV 47.67 ± 6.30% and 45.42 ± 10.86%. In the combined condition, immediate post-burpee heart rate was 183.58 ± 6.08 beats·min^−1^ (*n* = 12; range 172–190).

### 3.5. Physiological and Neuromuscular Outcomes

No significant overall condition effects were detected for HRend, HR60, HRR60 or SpO_2_, or for baseline-adjusted handgrip endurance ratio and stability error in either hand (all physiological/neuromuscular-family FDR q ≥ 0.422; Table 8). Complete model outputs, pairwise comparisons and sensitivity analyses for all outcomes are provided in Supplementary File S2.

## 4. Discussion

### 4.1. Fatigue Protocols and Shooting Performance

The sequential combined fatigue protocol was associated with lower four-minute shooting accuracy than control and the mental protocol alone, after accounting for period and assigned sequence. These comparisons persisted across the sensitivity analyses. Similar observed attempt volumes indicate that the main performance pattern concerned the proportion of successful shots within the timed task. The physical-versus-control dynamic-shooting contrast and the free-throw finding were more dependent on model choice.

At T1, paired condition comparisons showed higher mental fatigue ratings after the mental and combined protocols and higher physical ratings after the physical and combined protocols than after control. T1 captures the immediate subjective response to the intervention, whereas T2 follows the full assessment battery. The later increases in mental ratings after physical and physical ratings after mental, therefore, cannot be attributed to the assigned task alone. Immediate post-burpee heart rate further characterized the cardiovascular demand of the combined protocol. Enoka and Duchateau emphasize that objective performance measures do not directly quantify the intensity of the subjective fatigue symptom [39]. Here, the ratings describe reported fatigue, whereas shooting and vigilance outcomes describe performance responses to the protocols. The shooting result describes performance following the complete protocol; it does not identify an isolated neuromuscular or cognitive mechanism.

Previous basketball studies have linked cognitive fatigue protocols and sport-specific film exposure to changes in shooting [15], with variation across protocols and player characteristics [11,13]. The present comparison extends that work to a brief physical challenge immediately following sustained cognitive activity. Unlike the original SST, progression required two total, rather than two consecutive, successful shots [29]. This less restrictive criterion linked completed spots directly to the number of successful shots, so these counts should not be treated as independent evidence. Progression remained dependent on shooting success; consequently, the distribution of attempts across positions may have varied between conditions. Overall shooting accuracy, therefore, represents performance within this adapted task.

Free throws and dynamic shooting also differed in their assessment demands. The 20 free throws were attempted from a fixed position, whereas the four-minute task required repeated movement between positions. The shorter free-throw series provided fewer attempts per player. These differences should inform comparison of the outcomes, but do not establish why the free-throw finding was model-sensitive.

Combined fatigue protocols need not produce uniform effects across tasks. Mortimer et al. reported numerically better throwing and endurance performance in the combined than in the mental-only group; the endurance contrast was not statistically significant [17]. Their duration-matched cognitive/control phase followed by burpees or rest provides a precedent for sequential administration; our task and timing differed. Other studies have used simultaneous cognitive–physical tasks [16] or combined sleep restriction and cognitive loading in basketball [40]. The non-significant factorial interaction leaves uncertainty about additivity on the fitted scales; the lower combined-condition mean alone is insufficient to establish synergy.

### 4.2. Cognitive and Secondary Outcomes

Mental and combined protocols were associated with longer PVT-B reaction time and lower application-reported accuracy than control. The principal control comparisons persisted after session-specific baseline adjustment and in the sensitivity analyses. More lapses after combined than control provided additional evidence of poorer vigilance. The CV result was less consistent across inferential approaches.

PVT-B was administered after shooting, handgrip and MOT, so these findings characterize vigilance at the end of the experimental session. MOT outcomes did not reach significance after cognitive-family correction. Its reaction-time result was also sensitive to the finite-sample analysis. PVT-B and the application’s successive-target tracking task involve different response demands. Application-reported tracking-task latency should be interpreted alongside accuracy and the specific task configuration, as tracking paradigms and expertise-related outcomes vary across studies [41,42,43,44,45].

HRR60 characterizes cardiovascular recovery after the common shooting test [46], rather than the immediate response to the burpee intervention. Its timing, therefore, limits inference about cardiovascular differences immediately after the assigned protocols. Handgrip indices describe force control during a subsequent sustained upper-limb contraction, and are specific to that task and muscle group. They do not directly measure whole-body fatigue or the mechanics of shooting. The absence of statistically significant condition differences in these measures does not establish equivalent physiological or neuromuscular responses.

### 4.3. Practical Implications

The findings support recording shooting accuracy alongside attempt volume when evaluating performance after mental and physical fatigue protocols. In the tested sequence, similar numbers of attempts accompanied fewer successful shots. Whether this pattern transfers to competition, different exercise intensities or other cognitive tasks, requires direct field investigation.

### 4.4. Limitations

The sample of 12 male players from three regional-league clubs limits precision and generalizability. The large-effect assumption underlying the MAKE_4_MIN power calculation limits sensitivity to smaller effects, particularly for interactions and secondary outcomes. Three players per sequence restrict inference about sequence effects. The cyclic Latin square balanced periods, but not all preceding-condition pairs; period adjustment and design diagnostics therefore leave uncertainty about carryover and condition-specific learning.

Task-phase durations were not matched: the physical condition lasted approximately 7.5 min, including 6 min of pre-exercise rest and 90 s of burpees, compared with 30 min for control and mental and 31.5 min for combined. Comparisons involving the physical condition, therefore, cannot separate the effects of protocol content from those of unequal elapsed time. The findings should be interpreted as comparisons of the specific protocols used. Only the AX-CPT-before-burpee order was tested, so the effects of component order and proximity could not be isolated. Dynamic shooting followed 20 free throws, allowing some recovery before that assessment. Exercise can also modify responses to preceding cognitive activity [47,48], so reverse or concurrent arrangements may produce different results. Documentary engagement and participant expectations may influence subjective responses [49,50].

Burpee repetitions were not recorded, and immediate post-burpee heart rate was available only for the combined condition, limiting comparisons of exercise load between physical and combined protocols. Sleep, dietary intake and individual training duration and load were not monitored, and may have contributed to between-session variability. The exact interval from task completion to the start of dynamic shooting was also not recorded; therefore, the extent of recovery before this assessment could not be quantified.

The adapted shooting task requires further reliability evaluation. Study-specific subjective scales, application-specific cognitive scoring and the low-frequency video-derived handgrip indices also constrain interpretation beyond the procedures used. The mechanisms underlying the shooting response remain uncertain.

## 5. Conclusions

The sequential combined protocol—30 min AX-CPT followed by 90 s maximal burpees—was associated with lower four-minute shooting accuracy than control and the mental protocol alone. Mental and combined protocols were associated with slower PVT-B responses and lower application-reported accuracy than control, and combined was associated with more lapses. Free-throw and PVT-B variability findings were model-sensitive. MOT and physiological/neuromuscular outcomes did not reach significance after their respective domain corrections. The conclusions apply to this cognitive-then-physical procedure and the subsequent assessment sequence.

## Figures and Tables

**Table 1:** Participant characteristics.

Characteristic	*n*	Mean ± SD	95% CI
Age (years)	12	19.00 ± 1.04	18.34–19.66
Height (cm)	12	190.42 ± 8.06	185.29–195.54
Body mass (kg)	12	89.08 ± 17.78	77.78–100.38
BMI (kg·m^−2^)	12	24.34 ± 2.94	22.48–26.21

Values summarize the 12 players, once each. Confidence intervals are t-based intervals for the mean. BMI, body mass index; CI, confidence interval; SD, standard deviation.

**Table 2:** Shooting outcomes by condition.

Outcome	Control	Physical	Mental	Combined
Dynamic accuracy (%) [95% CI]	48.30 ± 12.85 [40.14, 56.46]	39.80 ± 9.99 [33.45, 46.15]	42.68 ± 12.82 [34.54, 50.82]	33.17 ± 9.66 [27.04, 39.31]
Made shots (*n*)	23.50 ± 7.26	19.42 ± 6.26	20.67 ± 6.76	16.25 ± 5.46
Missed shots (*n*)	24.75 ± 5.89	28.75 ± 4.97	27.33 ± 5.87	32.17 ± 3.66
Total attempts (*n*)	48.25 ± 4.75	48.17 ± 5.94	48.00 ± 5.39	48.42 ± 3.63
Completed spots (*n*)	11.50 ± 3.66	9.58 ± 3.06	10.00 ± 3.41	7.92 ± 2.61
Free-throw accuracy (%) [95% CI]	52.08 ± 19.94 [39.42, 64.75]	50.00 ± 12.06 [42.34, 57.66]	45.42 ± 13.39 [36.91, 53.93]	39.17 ± 13.79 [30.41, 47.93]

Values are participant-level means ± SD; accuracy rows include 95% CIs for the mean. Control, documentary; physical, 90 s burpees after 6 min rest; mental, 30 min AX-CPT; combined, AX-CPT followed by burpees. Completed spots are derived from two total makes per spot. Overall condition tests: dynamic accuracy, *p* < 0.001, q < 0.001; Four-minute made shots (MAKE_4_MIN), *p* = 0.002, q = 0.003; free-throw accuracy, *p* = 0.021, q = 0.028; attempts, *p* = 0.992, q = 0.992. False discovery rate (FDR) q values use the four-outcome basketball family. Adjusted dynamic-shooting accuracy contrasts are reported in Table 3.

**Table 3:** All condition contrasts for four-minute shooting accuracy.

Contrast	Adjusted Difference in pp [95% CI]	Holm *p*	Adjusted Odds Ratio [95% CI]	Holm *p*
Physical vs. Control	−8.50 [−15.21, −1.79]	0.052	0.70 [0.55, 0.89]	0.012
Mental vs. Control	−5.62 [−12.33, 1.09]	0.202	0.79 [0.63, 1.00]	0.103
Combined vs. Control	−15.13 [−21.84, −8.42]	<0.001	0.53 [0.41, 0.67]	<0.001
Mental vs. Physical	2.88 [−3.83, 9.59]	0.400	1.13 [0.89, 1.44]	0.304
Combined vs. Physical	−6.63 [−13.34, 0.08]	0.159	0.75 [0.59, 0.96]	0.064
Combined vs. Mental	−9.51 [−16.22, −2.80]	0.027	0.66 [0.52, 0.85]	0.004

Contrasts compare the first named condition with the second; condition definitions are given in Table 2. Percentage-point (pp) differences are from the participant-intercept linear sensitivity model; odds ratios (ORs) are from the binomial model. Both include condition, period and sequence. CIs are approximate pointwise 95% Wald intervals; Holm adjustment covers six contrasts within each model.

**Table 4:** Cognitive outcomes by condition.

Outcome	Control	Physical	Mental	Combined	*p*	FDR q
PVT-B reaction time	254.75 ± 25.64	264.58 ± 24.28	279.75 ± 29.27	278.67 ± 39.39	0.006	**0.020**
PVT-B accuracy	94.92 ± 2.50	88.67 ± 10.62	85.33 ± 10.32	85.25 ± 9.77	<0.001	**0.002**
PVT-B lapses	4.17 ± 2.44	7.25 ± 5.12	8.17 ± 5.31	10.67 ± 5.30	0.011	**0.020**
PVT-B CV	25.17 ± 9.18	46.92 ± 29.71	42.75 ± 18.99	51.50 ± 28.40	0.012	**0.020**
MOT reaction time	822.25 ± 100.10	761.67 ± 129.24	844.83 ± 134.02	826.92 ± 147.75	0.048	0.060
MOT accuracy	59.17 ± 9.08	63.58 ± 11.43	53.42 ± 14.68	61.00 ± 12.31	0.057	0.060
MOT CV	47.50 ± 11.10	52.00 ± 8.77	54.50 ± 8.23	53.50 ± 7.59	0.060	0.060

Values are post-session means ± SD; MOT was assessed once after the protocol. Condition definitions are given in Table 2. All models include condition, period and sequence; PVT-B models additionally adjust for the matching session baseline. Overall *p* values are from likelihood-ratio tests. FDR q values use the seven-outcome cognitive family; bold q values indicate significance at 0.05. Reaction times are in ms, accuracy and coefficient of variation (CV) in %, and lapses in counts. Adjusted PVT-B reaction-time and accuracy contrasts are reported in Table 5; MOT reaction-time contrasts are reported in Table 6.

**Table 5:** Adjusted condition contrasts for post-session PVT-B outcomes.

Contrast	RT Difference ms [95% CI]	Holm *p*	Accuracy Difference pp [95% CI]	Holm *p*
Physical vs. Control	8.30 [−9.19, 25.79]	0.705	−3.73 [−9.86, 2.41]	0.467
Mental vs. Control	24.32 [7.57, 41.07]	0.027	−9.28 [−15.17, −3.40]	0.010
Combined vs. Control	23.69 [7.10, 40.27]	0.027	−12.39 [−18.57, −6.22]	<0.001
Mental vs. Physical	16.02 [−0.84, 32.88]	0.250	−5.55 [−11.63, 0.52]	0.220
Combined vs. Physical	15.39 [−1.85, 32.63]	0.250	−8.67 [−15.57, −1.76]	0.056
Combined vs. Mental	−0.63 [−17.28, 16.02]	0.941	−3.11 [−9.35, 3.13]	0.467

Estimates are from participant-intercept linear mixed models with condition, categorical period, assigned sequence and the corresponding session baseline. Condition definitions are given in Table 2. Positive reaction-time differences indicate slower responses; negative accuracy differences indicate lower application-reported accuracy. CIs are approximate pointwise 95% Wald intervals; Holm adjustment covers six contrasts within each outcome. RT, reaction time.

**Table 6:** Adjusted condition contrasts for post-session MOT reaction time.

Contrast	Adjusted RT Difference, ms [95% CI]	Holm *p*
Physical vs. Control	−60.58 [−124.65, 3.48]	0.255
Mental vs. Control	22.58 [−41.48, 86.65]	1.000
Combined vs. Control	4.67 [−59.40, 68.73]	1.000
Mental vs. Physical	83.17 [19.10, 147.23]	0.066
Combined vs. Physical	65.25 [1.19, 129.31]	0.230
Combined vs. Mental	−17.92 [−81.98, 46.15]	1.000

Estimates are from a participant-intercept linear mixed model with condition, categorical period and assigned sequence. Positive differences indicate longer application-reported reaction times in the first named condition. CIs are pointwise 95% Wald intervals; Holm adjustment covers all six contrasts. The overall condition test belongs to the seven-outcome cognitive FDR family.

**Table 7:** Subjective fatigue ratings by condition and assessment time.

Rating	Condition	T0	T1	T2
Mental	Control	1.58 ± 0.51 ^a^	1.83 ± 0.58 ^a^	2.17 ± 0.83 ^a^
Mental	Physical	1.92 ± 1.08 ^a^	2.33 ± 1.23 ^a^	4.00 ± 1.71 ^b^*^†^
Mental	Mental	1.67 ± 0.78 ^a^	6.50 ± 2.02 ^b^*	7.08 ± 1.73 ^c^*
Mental	Combined	1.42 ± 0.79 ^a^	7.00 ± 1.35 ^b^*	8.08 ± 1.16 ^d^*
Physical	Control	1.50 ± 0.67 ^a^	2.00 ± 0.74 ^a^	2.58 ± 1.24 ^a^
Physical	Physical	2.25 ± 1.14 ^a^	5.17 ± 1.11 ^b^*	6.08 ± 1.51 ^bc^*
Physical	Mental	1.75 ± 0.87 ^a^	2.42 ± 0.79 ^a^	4.58 ± 1.93 ^ab^*^†^
Physical	Combined	1.75 ± 0.87 ^a^	6.25 ± 2.09 ^b^*	7.67 ± 1.61 ^c^*

Values are mean ± SD on study-specific 1–10 fatigue scales (*n* = 12). T0, before the assigned protocol; T1, immediately after the protocol; T2, after the assessment battery. Within each rating domain and time point, conditions with no shared superscript letter differ significantly. Within each condition and rating domain, * indicates a difference from T0 and ^†^ a difference from T1. All marked differences have Holm-adjusted *p* < 0.05; adjustments cover 18 between-condition comparisons and 12 within-condition time comparisons separately, for each rating domain. Shared letters indicate no statistically significant difference, not equivalence. Control, documentary; physical, 90 s burpees after 6 min rest; mental, 30 min AX-CPT; combined, AX-CPT followed by burpees. Condition × time interactions (Greenhouse–Geisser corrected; Holm adjustment across the two rating domains): mental, F(3.39, 37.31) = 32.97, ηp^2^ = 0.750; physical, F(3.95, 43.41) = 11.36, ηp^2^ = 0.508; both Holm *p* < 0.001.

**Table 8:** Cardiovascular and neuromuscular outcomes by condition.

Outcome	Control	Physical	Mental	Combined	*p*	FDR q
HRend	159.42 ± 25.45	159.33 ± 21.21	164.00 ± 19.84	164.58 ± 17.07	0.554	0.667
HR60	118.83 ± 18.49	111.67 ± 15.86	117.50 ± 10.21	116.50 ± 8.52	0.248	0.497
HRR60	40.58 ± 15.47	47.67 ± 9.68	46.50 ± 15.39	48.08 ± 12.00	0.158	0.422
SpO_2_	98.17 ± 0.72	98.17 ± 0.72	97.83 ± 0.83	98.00 ± 0.74	0.584	0.667
Dominant grip endurance ratio	1.015 ± 0.069	0.979 ± 0.031	0.998 ± 0.025	0.993 ± 0.073	0.505	0.667
Non-dominant grip endurance ratio	1.004 ± 0.045	0.995 ± 0.039	1.004 ± 0.029	1.001 ± 0.030	0.794	0.794
Dominant grip stability error	13.42 ± 8.44	7.58 ± 5.41	8.37 ± 5.13	11.56 ± 7.11	0.084	0.422
Non-dominant grip stability error	7.69 ± 2.70	9.70 ± 6.97	7.61 ± 3.10	8.90 ± 4.47	0.137	0.422

Values are post-session means ± SD; cardiovascular measures were end-only. Condition definitions are given in Table 2. All models include condition, period and sequence; handgrip models additionally adjust for the matching session baseline. Overall *p* values are from likelihood-ratio tests. FDR q values use the eight-outcome physiological/neuromuscular family. Heart-rate measures are in beats·min^−1^, SpO_2_ and stability error in %, and endurance measures in ratios. Peripheral oxygen saturation, SpO_2_.

## Data Availability

The deidentified session-level outcomes and allocation information are provided in Supplementary File S1. Reproducible analysis code and numerical results are provided in Supplementary File S2.

## Supplementary Materials

The following supporting information can be downloaded at: https://www.mdpi.com/article/10.3390/life16101620/s1, Supplementary File S1 contains deidentified session-level outcomes, participant codes, experimental periods, assigned sequences, shooting component counts, a data dictionary and scoring definitions. Supplementary File S2 contains reproducible analysis code, complete condition tests and contrasts and sensitivity results.
